# Cost of seeking care for tuberculosis since the implementation of universal health coverage in Indonesia

**DOI:** 10.1186/s12913-020-05350-y

**Published:** 2020-06-03

**Authors:** Ahmad Fuady, Tanja A. J. Houweling, Muchtaruddin Mansyur, Erlina Burhan, Jan Hendrik Richardus

**Affiliations:** 1 Department of Public Health, Erasmus MC, University Medical Center Rotterdam, Rotterdam, The Netherlands; 2 Department of Community Medicine, Faculty of Medicine, Universitas Indonesia, Jakarta, Indonesia; 3 Department of Respiratory and Pulmonology, Persahabatan Hospital – Faculty of Medicine, Universitas Indonesia, Jakarta, Indonesia

**Keywords:** Tuberculosis, Costs, Care-seeking behaviour, Universal health coverage, Indonesia

## Abstract

**Background:**

Although tuberculosis (TB) patients often incur high costs to access TB-related services, it was unclear beforehand whether the implementation of universal health coverage (UHC) in Indonesia in 2014 would reduce direct costs and change the pattern of care-seeking behaviour. After its introduction, we therefore assessed TB patients’ care-seeking behaviour and the costs they incurred for diagnosis, and the determinants of both.

**Methods:**

In this cross sectional study, we interviewed adult TB patients in urban, suburban, and rural districts of Indonesia in July–September 2016. We selected consecutively patients who had been treated for TB in primary health centers for at least 1 month until we reached at least 90 patients in each district. After establishing which direct and indirect costs they had incurred during the pre-diagnostic phase, we calculated the total costs (in US Dollars). To identify the determinants of these costs, we applied a general linear mixed model to adjust for our cluster-sampling design.

**Results:**

Ninety-three patients of the 282 included in our analysis (33%) first sought care at a private clinic. The preference for such clinics was higher among those living in the rural district (aOR 1.88, 95% CI 0.85–4.15, *P* = 0.119) and among those with a low educational level (aOR 1.69, 95% CI 0.92–3.10, *P* = 0.090). Visiting a private clinic as the first contact also led to more visits (β 0.90, 95% CI 0.57–1.24, *P* < 0.001) and higher costs than first visiting a Primary Health Centre, both in terms of direct costs (β = 16.87, 95%CI 10.54–23.20, *P* < 0.001) and total costs (β = 18.41, 95%CI 10.35–26.47, *P* < 0.001).

**Conclusion:**

Despite UHC, high costs of TB seeking care remain, with direct medical costs contributing most to the total costs. First seeking care from private providers tends to lead to more pre-diagnostic visits and higher costs. To reduce diagnostic delays and minimize patients’ costs, it is essential to strengthen the public-private mix and reduce the fragmented system between the national health insurance scheme and the National TB Programme.

## Background

Studies in Asian countries suggest that the private healthcare sector has the potential to play an important role in national and global tuberculosis (TB) control [1–4]. Many studies have shown that patients initially visit private providers to relieve them from TB-related symptoms [5]. In Indonesia, many patients also prefer first to seek care from private providers [4]. However, private providers often fail to comply with TB practice guidelines, including those for the screening and diagnosis of TB cases [4, 6]. In the pre-diagnostic phase, failure to comply with TB practice guidelines may lead to missed TB cases. Patients who continue to have TB related symptoms then have many healthcare visits [7], leading to diagnostic delays and high costs during seeking care [8, 9]. This eventually worsens disease prognosis at the individual level, increases costs in the household level, and spreads TB in the community [8, 10].

In 2014, Indonesia started implementing a universal health coverage (UHC) scheme, also called the Indonesian National Health Insurance scheme (*Jaminan Kesehatan Nasional*, JKN), to ensure people’s access to healthcare. This insurance scheme is managed by the Social Security Agency for Health *(Badan Penyelenggara Jaminan Sosial Kesehatan*, BPJS-K) and covers all essential care services, including TB diagnostic tests through healthcare providers that are linked to the BPJS-K [9, 11]. In the first 4 years after the JKN was implemented, the number of private providers linked to the BPJS-K – clinics and solo practices alike – increased substantially, from 6369 private providers to 11,507 [12].

However, BPJS-K is not directly linked to the network of the Indonesian National Tuberculosis Program (NTP), which are national level government boards that are responsible for TB control. Private providers that are linked to the BPJS-K are therefore also not necessarily linked to the NTP. Responding to this problem, the NTP launched a guideline of TB care to coordinate the care with the BPJS-K [13]. Although there has been no direct linkage between the NTP and BPJS-K, the private providers that are linked with BPJS-K can conduct the TB tests in their laboratories, if available, or can refer suspected TB patients for diagnostic tests to a BPJS-K-linked facility [13]. All consultation and diagnostic test fees are covered by the BPJS-K. If the suspected patient has received the final TB diagnosis, the private providers can refer the patient to the NTP-linked facilities to receive free TB treatment. As most TB patients seek initial care with a private provider [6], this coordinated scheme between BPJS-K and the NTP is assumed to reduce patient’s direct costs during the pre-diagnostic phase.

Nevertheless, there is no evidence available on the costs incurred during the pre-diagnostic phase of TB and TB patients’ care seeking behaviour since the implementation of UHC in Indonesia. It is therefore unclear to which extent UHC has saved patients from high costs during this phase. The aims of this study are to assess the costs incurred before diagnosis by patients seeking care for TB after the implementation of UHC in Indonesia, and to assess care-seeking behaviour of TB patients in this period on the basis of the first contact facility and the number of healthcare visits.

## Methods

### Study design and setting

To assess patients’ TB care-seeking behaviour and the costs they incurred during the pre-diagnostic phase, we conducted a cross-sectional study that was a part of a larger study on measuring catastrophic costs in Indonesia [9]. In this study, we interviewed patients who had undergone TB treatment in PHCs (i.e. public facilities for primary-level care). We selected three districts in Java, the most populous island of Indonesia, purposively to represent urban (Jakarta), suburban (Depok), and rural (Tasikmalaya) areas of Indonesia.

### Study population

We included all subjects who met the inclusion criteria of our main study, which were adult patients who had received TB treatment in a PHC for at least 1 month. We excluded extra-pulmonary cases, as these are diagnosed using different methods that may result in bias on pre-diagnostic costs.

### Sampling method

The sample size and sampling methods in this study followed that of our larger study, which assumed an incidence of catastrophic costs due to TB of 20–30% [9]. We used a sample size formula for a cross sectional study (Zα^2^.p.q/d^2^) with assumptions of a power of 0.90 and an error of 0.05. With a ratio of TB patients of 1:1:1 in each district, we required 90 patients per district. In each district, we included all the PHCs that were linked to the Indonesian NTP in our sampling framework, and then randomly selected five PHCs per district. We chose only PHCs since most of TB patients were treated in PHCs. In each PHC, we selected consecutively adult TB patients until we reached at least 90 patients per district. If the sample size was not reached, we randomly selected additional PHCs until we obtained at least 90 patients per district. In total, 19 PHCs were included in our study.

### Data collection

All patients were interviewed by four medical students and six public health graduates we had recruited and trained for the purpose. As a part of our main study on measuring catastrophic costs, the interview was conducted during the TB treatment phase – 48% of patient in their intensive phase and 52% of patients in their continuous phase [9]. To interview the patients and/or the drug observer who accompanied each patient as a direct observation of treatment (DOT) supporter, these interviewers used the adapted and validated Bahasa Indonesia version of the Tool to Estimate Patient Costs [14].

Retrospectively, each respondent listed each healthcare facility he or she had visited between developing TB-related symptoms (e.g., chronic cough, bloody cough, weight loss, or night sweating) and the establishment of their TB diagnosis [14]. We also calculated the number of healthcare visits and costs incurred during the pre-diagnostic phase, and then assessed the determinants of the number of visits and costs.

### Care seeking behavior

First, to assess patients’ care-seeking behaviour, we established the nature of their first contact facility, i.e., a primary health centre (PHC); a private clinic (whether a solo practice or part of a multiple practice); a public hospital; a private hospital; or ‘other’, such as a pharmacy, a practitioner of alternative medicine, or a *mantri* (i.e., a registered nurse practicing as an unauthorized physician). We then assessed whether the first contact facility was associated with the district (urban/suburban/rural), household income level (poor/non-poor household), educational level (low/middle-high), health insurance status (being covered/not being covered by the BPJS-K scheme), and formal employment (yes/no).

### Number of visits

Second, we calculated the number of healthcare visits made during the pre-diagnostic phase. To assess the determinants of the number of visits, we also included first contact as an independent variable, together with the other independent variables.

### Costs of care seeking

Third, we asked patients the details of all the types of cost they had incurred in each facility visited during the pre-diagnostic phase, i.e., direct medical costs, direct non-medical costs, and indirect costs related to seeking care for their TB-related symptoms. The definitions of cost items used in this study conformed with the WHO handbook on TB costs survey [15]. The direct medical costs included all out-of-pocket (OOP) payments to the healthcare facilities for medical fees, such as administrative charges and the cost of drugs, laboratory analyses, or X-ray examinations. The direct non-medical costs included the cost of food and travel for patients and/or their guardian during their visits to healthcare facilities. The indirect costs consisted of loss of income incurred by the patients and their guardians on their visits to healthcare facilities. The total costs were the sum of direct medical costs, direct non-medical costs, and indirect costs. All costs were provided in Indonesian Rupiahs (IDR) and then converted to US dollars using the World Bank’s average exchange rate for 2015 (1 USD = 13,389.41 IDR). We then assessed whether the total costs, direct costs, and indirect costs were associated with the first contact, district, household income level, educational level, health insurance status, and formal employment.

### Data management and analysis

Data were double-entered into Microsoft Excel 2010 and EpiInfo version 7 (CDC, Atlanta) and then exported to IBM SPSS 21 for data analysis. Number of visits and costs data were displayed as means and their 95% confidence intervals (CIs) while categorical variables as numbers and proportions (%). To examine the determinants of the patients’ first contact facility, the number of visits, and their costs during the pre-diagnostic phase, we applied the generalized linear mixed models (GLMMs) with random effects to adjust for our cluster sampling design (19 PHCs), since we selected the PHCs randomly. We assessed the goodness of fit for models by examining the Akaike corrected and the Bayesian information criterion provided by SPSS. To analyse the determinants of the first health-service contact, we estimated crude odds ratios (cORs), adjusted odds ratios (aORs), and their 95% CIs with a target distribution of multinomial regression. To analyse the determinants of number of visits and costs incurred by patients and their family, we established crude and adjusted GLMM regression coefficients (β) and their 95% CIs with a target distribution of linear regression.

### Ethical issues

Before the interview, we provided all respondents with a written and oral explanation of the study. Only those who had signed an informed consent form were interviewed. Before the study, we received ethical clearance from the Ethical Committee at the Faculty of Medicine of Universitas Indonesia–Cipto Mangunkusumo Hospital, Jakarta, Indonesia (No. 416/UN2.F1/ETIK/VI/2016).

## Results

We included the interviews of 282 patients. Whereas a PHC had been the first point of diagnostic care-seeking for 45% of them, 33% had first sought care at a private clinic, 11% at a public hospital, 7% at a private hospital, and 4% at a pharmacy, practitioner of alternative medicine, or other healthcare provider. (Table 1) A majority of patients (58%) had been diagnosed at a PHC. The remainder had been diagnosed at a private clinic (15%), public hospital (18%), or private hospital (9%) before they were referred to PHCs for TB treatment.

**Table 1:** First point of care and place of TB diagnosis of a sample of 282 TB patients

Healthcare service	First point of care		Place of diagnosis	
	N	%	n	%
Primary health centre	127	45	164	58
Private clinic	93	33	43	15
Public hospital	32	11	50	18
Private hospital	20	7	25	9
Other health provider^a^	10	4	0	0

^a^Pharmacy, practitioner of alternative medicine, *mantri*, or other health provider

**Fig. 1:**
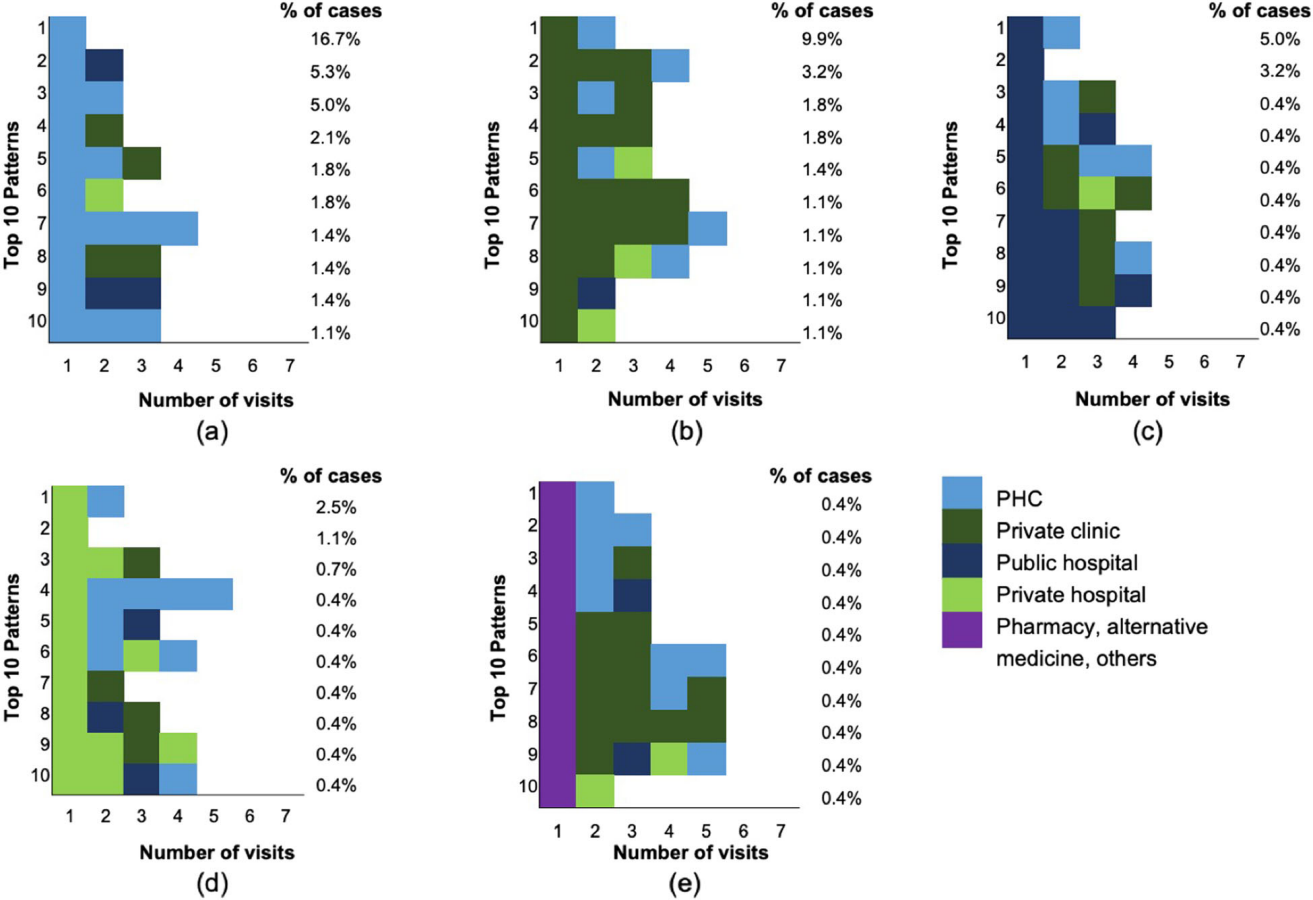
The care-seeking pattern of TB patients onwards of their first contact. The figure shows the top 10 care-seeking patterns that followed the first contact with each of the following: **a** a PHC, **b** a private clinic, **c** a public hospital, **d** a private hospital, and **e** a pharmacy, practitioner of alternative medicine, or other health provider. Each coloured block indicates the type of healthcare provider visited. Each rightmost box indicates the provider where the TB diagnosis was confirmed. The percentages to the right of each graph are the percentages of each patterns. Eighteen percents of patterns are not captured in these graphs

Figure 1 shows the most common care-seeking patterns identified in this study. Seventeen percent of patients had been diagnosed during their first visit at PHC. This was higher than the percentage of patients who had been diagnosed during their first visit to any of the other healthcare settings. All patients who had first sought care at a pharmacy, practitioner of alternative medicine or other healthcare provider had moved to another provider – usually a private clinic – for their second visit.

Table 2 shows that in the urban and suburban districts, more patients initially sought care at a PHC than at another healthcare facility. In contrast, among patients with low education level, the proportion of those who initially sought care at a private clinic was higher than at another healthcare facility.

**Table 2:** Distribution of patients by point of first contact

Characteristics	Primary health centre *N (%)*	Private clinic *N (%)*	Public hospital *N (%)*	Private hospital *N (%)*	Other health provider^a^ *N (%)*	Total
*Number of patients*	*127 (45)*	*93 (33)*	*32 (11)*	*20 (7)*	*10 (4)*	*282 (100)*
Districts						
Urban	51 (54)	26 (27)	11 (12)	4 (4)	3 (3)	95 (100)
Suburban	40 (44)	25 (28)	10 (11)	13 (14)	2 (2)	90 (100)
Rural	36 (37)	42 (43)	11 (11)	3 (3)	5 (5)	97 (100)
Household income level^b^						
Non-Poor	51 (48)	30 (28)	12 (11)	13 (12)	1 (1)	107 (100)
Poor	76 (43)	63 (36)	20 (11)	7 (4)	9 (5)	175 (100)
Education level^c^						
Middle-High	89 (49)	50 (27)	22 (12)	15 (8)	7 (4)	183 (100)
Low	38 (38)	43 (43)	10 (10)	5 (5)	3 (3)	99 (100)
National health insurance						
Not covered	48 (44)	41 (37)	8 (7)	7 (6)	6 (5)	106 (100)
Covered	79 (46)	52 (30)	24 (14)	13 (8)	4 (2)	176 (100)
Workers in informal sectors						
No	41 (51)	25 (31)	7 (9)	5 (6)	3 (4)	81 (100)
Yes	86 (43)	68 (34)	25 (12)	15 (7)	7 (3)	201 (100)

^a^ Pharmacy, alternative medicine, *mantri*, and other health providers

^b^ household earning below 1.9 USD per capita per day was classified as a poor household

^c^ a patient who did not graduate from elementary school was classified as having low education level

Table 3 shows that more patients in the rural district preferred to seek care first at a private clinic (cOR 2.27, 95% CI 1.14–4.53, *P* = 0.020). Seeking inital care at a private provider rather than a PHC was also greater among patients with a low educational level than among those with a middle to high educational level (cOR 1.93, 95% CI 1.09–3.41, *P* = 0.024). Despite the borderline statistical significance of the result, multivariable analysis showed that the odds of seeking care at a private clinic rather than at a PHC was higher in the rural district (aOR 1.87, 95% CI 0.85–4.15, *P* = 0.119) and in patients with low education level (aOR 1.69, 95% CI 0.51–1.65, *P* = 0.090). There were no statistically significant differences regarding first contact preferences for the following: between patients with insurance and without insurance; between patients living in a poor household and a non-poor household; and between patients with and without formal work.

**Table 3:** Determinants of the first point of health-service contact (private clinic or primary health centre)

Characteristics	Private clinic N (%)	PHC^**a**^ N (%)	P	cOR (95% CI)	P	aOR (95% CI)
Districts						
Urban^a^	26 (27)	51 (54)		1		1
Suburban	25 (28)	40 (44)	0.557	1.24 (0.60–2.55)	0.462	1.32 (0.63–2.76)
Rural	42 (43)	36 (37)	0.020	2.27 (1.14–4.53)	0.119	1.88 (0.85–4.15)
Household income level‡						
Poor	63 (36)	76 (43)	0.355	1.32 (0.74–2.35)	0.824	1.07 (0.57–2.01)
Non-Poor^a^	30 (28)	51 (48)		1		1
Education level±						
Low	43 (43)	38 (38)	0.024	1.93 (1.09–3.41)	0.090	1.69 (0.92–3.10)
Middle to high^a^	50 (27)	89 (49)		1		1
National health insurance						
Covered	52 (30)	79 (46)	0.468	0.81 (0.46–1.43)	0.768	0.92 (0.51–1.65)
Not covered^a^	41 (37)	48 (44)		1		1
Workers in informal sectors						
Yes	68 (34)	86 (43)	0.364	1.32 (0.72–2.41)	0.284	1.40 (1.76–2.58)
No^a^	25 (31)	41 (51)		1		1

^a^Reference category; ‡ household earning below 1.9 USD per capita per day was classified as a poor household; ± a patient who did not graduate from elementary school was classified as having low education level

The average number of visits during the pre-diagnostic phase was 2.56 (95% CI 2.41–2.71). Patients who first sought care at private clinics and other health care providers had more visits until their diagnosis was confirmed than patients who first visited a PHC (aβ 0.90, 95% CI 0.57–1.24, *P* < 0.001 for private clinics and aβ 1.77, 95% CI 0.97–2.57, *P* < 0.001 for other health care providers). (Table 4) There were no statistically significant differences in the number of visits regarding the following determinants: district, household income level, educational level, having insurance, and employment status.

**Table 4:** Determinants of the number of healthcare visits during the pre-diagnostic phase

Variables	No of visits mean (95% CI)	cβ (95% CI)	***P***	aβ (95% CI)	***P***
*Total number of visits*	*2.56 (2.41–2.71)*				
First contact facility					
Primary Health Centre	2.18 (1.96–2.41)	Ref		Ref	
Private clinic	3.06 (2.83–3.30)	0.89 (0.57–1.22)	< 0.001	0.90 (0.57–1.24)	< 0.001
Public hospital	2.13 (1.77–2.48)	− 0.07 (− 0.55–0.41)	0.766	− 0.08 (− 0.56–0.41)	0.750
Private hospital	2.65 (2.06–3.24)	0.44 (− 0.14–1.02)	0.140	0.39 (− 0.20–0.99)	0.194
Other health provider	3.9 (2.66–5.14)	1.70 (0.91–2.49)	< 0.001	1.77 (0.97–2.57)	< 0.001
District					
Urban	2.49 (2.18–2.63)	Ref		Ref	
Suburban	2.63 (2.37–2.90)	0.21 (−0.29–0.70)	0.406	0.17 (− 0.31–0.66)	0.484
Rural	2.56 (2.34–2.78)	0.11 (−0.39–0.62)	0.667	−0.05 (− 0.57–0.48)	0.861
Household income level^a^					
Poor	2.58 (2.38–2.77)	0.07 (−0.25–0.40)	0.656	0.02 (−0.34–0.31)	0.927
Non-poor	2.53 (2.29–2.78)	Ref		Ref	
Education level^b^					
Low	2.66 (2.39–2.92)	0.16 (−0.17–0.49)	0.336	0.14 (−0.22–0.43)	0.526
Middle-high	2.51 (2.32–2.70)	Ref		Ref	
National health coverage					
Covered	2.58 (2.37–2.79)	0.07 (−0.25–0.39)	0.673	0.18 (−0.13–0.49)	0.259
Not covered	2.53 (2.32–2.74)	Ref		Ref	
Workers in informal sectors					
Yes	2.55 (2.37–2.72)	−0.07 (−0.41–0.27)	0.692	−0.12 (− 0.44–0.21)	0.480
No	2.59 (2.29–2.90)	Ref		Ref	

β is the GLMM coefficient of the expected change in the number of visits compared to the reference category; *cβ* crude coefficient β; *aβ* adjusted coefficient β; *CI* confidence interval; *Ref* reference; *P* value of significance; ^a^ household earning below 1.9 USD per capita per day was classified as a poor household; ^b^ a patient who did not graduate from elementary school was classified as having low education level

The average total cost incurred during the pre-diagnostic phase was USD 22 (95% CI 18–26). It consisted mainly of direct costs (USD 16, 95% CI 13–19). (Table 5) Visiting a private clinic as the first point of contact led to statistically significantly higher costs than visiting a PHC as the first contact, both in terms of direct costs (β 16.87, CI 95% 0.54–23.20, *P* < 0.001) and of total costs (β 18.41, CI 95% 10.35–26.47, *P* < 0.001). Patients who visited a private hospital as the first contact also incurred statistically significantly higher direct costs (β 28.38, CI 95% 17.18–39.58, *P* < 0.001) and total costs (β 24.96, CI 95% 10.64–39.28, *P* = 0.001) than those visiting a PHC as the first contact. The direct medical costs incurred by patients who first sought care at private clinics (USD 21; 95% CI USD 15–28) and private hospitals (USD 32; 95% CI USD 17–48) were significantly higher than those who first sought care at PHCs (USD 5; 95% CI USD 3–7), while travel costs between private providers and PHCs did not differ significantly (See Additional file 1). Despite involving a higher number of visits, first visiting a pharmacy, practitioner of alternative medicine or other health provider did not lead to significantly higher costs than those incurred by patients who first sought care at a PHC. Between districts, insurance coverage, household income level, and education level, the differences in the total costs, direct costs, and indirect costs incurred were not statistically significant. Indirect costs during the pre-diagnostic phase were associated with employment status. In addition, patients who were formally employed had higher indirect costs than patients who did not have a job or were not formally employed (β 3.92, CI 95% 0.88–6.96, *P* = 0.012). No other variables determined the indirect costs.

**Table 5:** Determinants for cost of seeking care, in USD

Variables	Total costs			Direct costs			Indirect costs		
	Mean (95% CI)	β (95% CI)	***P***	Mean (95% CI)	β (95% CI)	***P***	Mean (95% CI)	β (95% CI)	***P***
*Costs*	*22 (18–26)*			*16 (13–19)*			*6 (4–7)*		
First contact facility									
Primary Health Centre	14 (10–17)	Ref		8 (6–10)	Ref		6 (4–8)	Ref	
Private clinic	32 (23–41)	18.41 (10.35–26.47)	< 0.001	25 (18–32)	16.87 (10.54–23.20)	< 0.001	7 (4–10)	1.75 (−1.44–4.95)	0.280
Public hospital	18 (9–27)	5.30 (−6.46–17.06)	0.376	13 (7–18)	4.98 (−4.22–14.18)	0.288	6 (1–10)	0.19 (− 4.48–4.85)	0.937
Private hospital	37 (22–52)	24.96 (10.64–39.28)	0.001	36 (20–51)	28.38 (17.18–39.58)	< 0.001	1 (0–3)	−3.50 (−9.19–2.18)	0.226
Other health provider	18 (7–29)	5.38 (−14.02–24.78)	0.586	14 (5–24)	6.83 (−8.40–22.06)	0.378	4 (−1–8)	−1.32 (− 8.99–6.36)	0.736
District									
Urban	23 (14–31)	Ref		16 (9–22)	Ref		7 (4–10)	Ref	
Suburban	20 (15–25)	−1.18 (−12.55–10.20)	0.839	16 (11–21)	1.81 (−6.89–10.50)	0.683	3 (2–5)	−3.23 (− 7.94–1.48)	0.178
Rural	23 (19–28)	1.80 (−9.79–13.39)	0.760	17 (13–20)	1.57 (− 7.22–10.36)	0.725	7 (5–9)	0.23 (− 4.62–5.08)	0.926
Household income level‡									
Poor	22 (17–28)	1.93 (−5.77–9.63)	0.622	17 (13–21)	1.59 (−4.57–7.74)	0.612	6 (4–7)	0.16 (−2.80–3.11)	0.918
Non-poor	21 (16–26)	Ref		15 (11–19)	Ref		6 (3–8)	Ref	
Education level±									
Low	22 (18–27)	0.42 (−7.40–8.24)	0.916	17 (13–20)	0.61 (−5.638–6.86)	0.848	6 (4–8)	−0.22 (−3.25–2.75)	0.869
Middle-high	22 (16–27)	Ref		16 (12–20)	Ref		6 (4–8)	Ref	
National health insurance									
Covered	20 (16–23)	−5.50 (−13.17–2.17)	0.159	14 (12–17)	−4.785 (− 10.89–1.32)	0.124	5 (4–7)	−0.64 (−3.59–2.32)	0.672
Not covered	26 (18–34)	Ref		19 (13–26)	Ref		7 (4–9)	Ref	
Workers in informal sectors									
Yes	23 (18–28)	3.26 (−4.82–11.34)	0.428	16 (12–20)	−0.52 (−7.04–6.00)	0.876	7 (5–9)	3.92 (0.88–6.96)	0.012
No	19 (15–24)	Ref		16 (12–20)	Ref		3 (2–4)	Ref	

Direct costs include those incurred by patients and/or their guardians during the pre-diagnostic phase for consultation, administrative fees, diagnostic tests (sputum and/or X-ray examination), food, and travel. Indirect costs include their income losses due to visits to healthcare facilities during the pre-diagnostic phase. Total costs are the sum of direct and indirect costs. β is the GLMM coefficient of the expected change in the number of visits compared to the reference category; *CI* confidence interval; *Ref* reference; *P* value of significance; ‡ Household earning below 1.9 USD per capita per day was classified as a poor household. ± A patient who did not graduate from elementary school was classified as having low education level

## Discussion

Our results show that patients still incur high costs while seeking TB care. Direct medical costs contributed most to the total costs of TB care seeking, despite the implementation of UHC in Indonesia. Most patients who were treated for TB in a PHC had started their care-seeking in private sector. The number of those who first sought care at a private clinic first was significantly greater among patients who lived in the rural district and among those with a lower educational level. Before being diagnosed with TB, patients who had first sought care at such clinics made more healthcare visits and had higher costs than those whose first point of contact was a PHC.

Rural patients’ preference for starting to seek care at a private clinic may have been affected by the greater distance they had to travel to a PHC. The number of PHCs in these areas is limited, the cost of transport to them is higher, and the waiting times can be long [16]. This encourages patients to visit any private healthcare provider – whether clinic, solo practice or other – that is closer to their house.

However, to have their diagnosis of TB confirmed, patients whose first point of contact was a private clinic needed more visits than those who first visited a PHC. This higher number of visits may have been due to the poorer TB-service readiness of private clinics, most of which – particularly private solo practices – do not have appropriate facilities for TB diagnostic tests, i.e., a laboratory for sputum smear examination [17]. To solve this problem, we suggest that most clinics use sputum smear fixation and deliver the preparation for diagnosis to a referral PHC or a clinic linked with the BPJS-K [13].

Our findings also suggest that seeking care first at a private clinic or private hospital led to significantly higher costs during the pre-diagnostic phase. Except when covered by health insurance, each visit to a private hospital is costly [18, 19]. This explains why, despite the limited number of visits, high costs were incurred at private hospitals. However, while the high cost of private clinics sometimes resulted from the high costs per visit, it also may be resulted from a high number of visits or from a combination of both.

Patients who first sought care at a pharmacy, practitioner of alternative medicine or other healthcare provider had a higher number of visits than those who started care-seeking at PHCs. However, this high number of visits did not lead to significantly higher costs for diagnosis. This may have been because the costs of simple, generic medicine in a pharmacy or of consultations for alternative medicine were low.

Despite the implementation of UHC in Indonesia, excessive visits and costs during the pre-diagnostic phase remain. Although this study did not compare the situation before and after the implementation of UHC, we showed that there were no significant differences in number of visits and costs between patients who were and were not covered by national health insurance.

Excessive visits and costs can result in diagnostic delays, potential catastrophic costs during treatment, and poor outcome [10, 20]. To prevent high number of visits and high costs, the integration between the national health insurance and the NTP, which is still fragmented, should be improved [21]. Currently, there has been no direct link between the national health insurance system and the NTP. Private providers – despite linked to the BPJS-K – often unaware of the national tuberculosis guidelines and of TB referral system under the NTP [4]. The current practice of TB current guidelines in private providers seems not optimal. There has been also lack of incentives from the national health insurance to improve the quality of TB care in private sector [22]. Therefore, comprehensive strategies are imperative. To solve the fragmented system in TB care, the national health insurance needs to develop a mechanism of incentives whereby private physicians and clinics can screen and diagnose TB cases accurately and refer the case to facility where TB diagnostic tests and treatment are fully covered. In its pay-for-performance criteria [22], for example, BPJS-K should include the quality of the TB services a clinic provides. A contract of service provision that is signed between the BPJS-K and private providers should consider the readiness of the TB services including the availability of diagnostic tests, the adherence with TB management guidelines, and prior attendance of TB training. In addition, the strategies should include efforts to increase patients’ awareness, to reduce stigma and discrimination, to improve TB diagnostic options, and to increase the number of PHCs in rural districts.

This study has two main limitations. The first is our collection of data from patients who had ended up at a PHC for TB treatment, and thus our exclusion of those who had had TB treatment from a private provider. This may have led us to overestimate PHCs as the first point of contact and to underestimate the number of pre-diagnostic visits and the costs incurred during the pre-diagnostic phase. It may also have led to a misleadingly high figure for the number of visits – by at least one visit – by patients whose first point of care had not been been a PHC, particularly if this visit had involved a healthcare provider that could not itself diagnose TB. The second limitation is that, since we relied on patients’ memory to obtain the information, the number of visits and the costs incurred may also have been affected by recall bias. A patient may not remember the frequency and cost of buying medicine in a drug store or pharmacy, and thereby underestimate the number of visits and the total pre-diagnostic costs.

The provision of TB services in Indonesia is similar to health-service delivery in other high TB-burden countries in Asia that need to improve their public-private mix. However, our findings require careful generalization before being applied to other countries or even to other regions of Indonesia. As this study was conducted only on the island of Java, it does not necessarily reflect the situation throughout Indonesia.

## Conclusion

Despite the UHC, high costs of TB seeking care remain, with direct medical costs contributing most to the total costs. The preference of people with TB first to seek diagnosis from a private provider rather than a PHC leads to more pre-diagnostic visits and higher costs. The UHC scheme alone is not enough to improve TB control and reduce patients’ costs. A comprehensive strategy is required to improve TB-related services in the private healthcare sectors. To reduce diagnostic delays and minimize patients’ costs, it is essential to reduce the fragmented system between the national health insurance scheme and the National TB Programme, to improve the quality of TB care in the private sector, and to improve the availability of PHCs, particularly in rural areas.

## Supplementary information

**Additional file 1.** Average pre-diagnostic costs according to the patient’s first point of contact, in USD, mean (95% CI)

## Data Availability

Data analysed during the current study available from the corresponding author on reasonable request.

## Abbreviations

BPJS-K: *Badan Penyelenggara Jaminan Sosial Kesehatan* (The Social Security Agency for Health)
CI: Confidence interval
IDR: Indonesian Rupiah
IQR: Interquartile range
NTP: National Tuberculosis Programme
PHC: Primary Health Centre
OR: Odds ratio
cORs: Crude Odds Ratio
aORs: Adjusted Odds Ratio
TB: Tuberculosis
UHC: Universal Health Coverage
USD: US Dollar
WHO: World Health Organization
