# Supplementary material for: Cost of seeking care for tuberculosis since the implementation of universal health coverage in Indonesia
Source: BMC Health Serv Res. 2020 Jun 3;20:502. doi: 10.1186/s12913-020-05350-y (PMC7271484; doi:10.1186/s12913-020-05350-y)
Supplement: Supplementary file 1 — Additional file 1. Average pre-diagnostic costs according to the patient’s first point of contact, in USD, mean (95% CI) [file 12913_2020_5350_MOESM1_ESM.docx]

**Appendix** Average pre-diagnostic costs according to the patient’s first point of contact, in USD, mean (95% CI)

|  | **Direct Medical Costs** | ***P*** | **Travel costs** | ***P*** | **Food costs** | ***P*** | **Total costs** | ***P*** |
| --- | --- | --- | --- | --- | --- | --- | --- | --- |
| PHC | 5.4 (3.3-7.4) |  | 1.8 (1.2-2.4) |  | 0.6 (0.4-0.8) |  | 14 (10-17) |  |
| Private clinics | 21.4 (14.6-28.1) | <0.001 | 2.7 (1.8-3.5) | 0.087 | 0.7 (0.4-1.0) | 0.424 | 32 (23-41) | <0.001 |
| Public hospitals | 8.9 (3.4-14.4) | 0.408 | 2.5 (1.5-3.4) | 0.274 | 1.1 (0.5-1.7) | 0.094 | 18 (9-27) | 0.376 |
| Private hospitals | 32.4 (17.3-47.5) | <0.001 | 2.6 (0.7-4.5) | 0.180 | 0.7 (-0.2-1.6) | 0.580 | 37 (22-52) | 0.001 |
| Others | 12.1 (2.1-22.2) | 0.366 | 2.1 (0.6-3.6) | 0.725 | 0.3 (-0.1-0.6) | 0.630 | 18 (7-29) | 0.586 |
